# Supplementary material for: Large mode volume integrated Brillouin lasers for scalable ultra-low linewidth and high power
Source: Nat Commun. 2025 Jul 11;16:6419. doi: 10.1038/s41467-025-61637-w (PMC12254478; doi:10.1038/s41467-025-61637-w)
Supplement: Supplementary file 1 — Supplementary Information [file 41467_2025_61637_MOESM1_ESM.pdf]

Supplementary Information: Large Mode Volume Integrated Brillouin Lasers for Scalable Ultra-Low Linewidth and High Power

Kaikai Liu[1], Karl D. Nelson[2], Ryan O. Behunin[3, 4], Daniel J. Blumenthal[1*]

[1]Department of Electrical and Computer Engineering, University of California Santa Barbara, Santa Barbara, CA, USA
[2]Honeywell Aerospace, Plymouth, MN, USA
[3]Department of Applied Physics and Materials Science, Northern Arizona University, Flagstaff, Arizona, USA
[4]Center for Materials Interfaces in Research and Applications (¡MIRA!), Northern Arizona University, Flagstaff, AZ, USA
*Corresponding author (danb@ucsb.edu)

Supplementary Note 1: 4-meter-coil resonator design and characterization

Table S1 summarizes the main characteristics of the stimulated Brillouin scattering (SBS) lasers such as output power, efficiency and fundamental linewidth. Normally due to cascaded emission, the SBS laser operates with the pump power of $P_{in} = 4P_{th}$ to achieve the minimum fundamental linewidth $\Delta\nu_{ST} = \mu(n_{th} + 1)/2\pi$, where μ is the cavity Brillouin gain rate per photon and n_{th} is the thermal occupation number of the acoustic mode, defined in Ref. [1]. With S2 suppression, the S1 output power scales with the square-root of pump power and thus the efficiency drops at high pump power. Whereas, with S3 suppression, the S1 output power scales linearly with the pump power.

Table S1 SBS laser in (1) cascaded, (2) S2-suppressed, (3) S3-suppressed emissions.

	Output power	Efficiency	Linewidth (*)
(1) Cascaded emission at $P_{in} = 4P_{th}$	$P_{S1} = \frac{Q_L^2}{Q_{ex}^2} 4P_{th}$	$\eta_{S1} = \frac{Q_L^2}{Q_{ex}^2}$	$\Delta\nu_{ST} = \frac{\mu(n_{th}+1)}{2\pi} \sim \frac{1}{L}$
(2) S2-suppressed emission	$P_{S1} = 4\frac{Q_L^2}{Q_{ex}^2}\left(\sqrt{P_{th}P_{in}} - P_{th}\right)$	$\eta_{S1} \cong 4\frac{Q_L^2}{Q_{ex}^2}\sqrt{P_{th}/P_{in}}$	$\Delta\nu_{ST} = \frac{\hbar\omega^3(n_{th}+1)}{4\pi Q_L Q_{ex} P_{S1}}$
(3) S3-suppressed emission	$P_{S2} = \frac{Q_L^2}{Q_{ex}^2}(P_{in} - 4P_{th})$	$\eta_{S2} \cong \frac{Q_L^2}{Q_{ex}^2}$	$\Delta\nu_{ST} = \frac{\hbar\omega^3(n_{th}+1)}{4\pi Q_L Q_{ex} P_{S2}}$

Table S1. Output power, conversion efficiency, and fundamental linewidth for (1) cascaded, (2) S2-suppressed, (3) S3-suppressed SBS lasers. P_{in} is the input pump power, P_{th} is the S1 threshold, Q_{ex} and Q_L are the cavity external coupling and loaded Qs, and n_{th} is the thermal occupation number of the acoustic mode. (*The optical mode thermal occupation number N_{th} is negligible at room temperature and neglected in the term of $(n_{th} + 1)$, $(n_{th} + N_{th} + 1) \cong (n_{th} + 1)$. If the SBS linewidth enhancement factor α is non-zero[2], the linewidth expressions can be multiplied by $(1 + \alpha^2)$. The derivation of these equations can be found in previous work[1,3,4].

The fundamental TE mode in the 6 μm by 80 nm waveguide shown in Fig. S1**a** can have a bending radius down to 1.0 mm without inducing significant bending loss, shown in Fig. S1**b**. The small

bending radius of this waveguide design enables packing the 4-meter-long coil waveguides in a small area of 1.2 cm by 1.2 cm, as shown in Fig. S1**c**. The 4-meter-coil resonator device is fiber pig-tailed using UHNA fibers for better matching with the waveguide mode, and packaged in a metal enclosure, shown by a photo of the device in Fig. S1**c**. The mode matching simulation on the fiber modes and the tapered waveguide mode at different tapering widths is shown in Fig. S2. The resonator bus waveguide uses a waveguide taper of 11 μm in this current design, and future designs will use 1.0 or 1.5 μm tapering width for better coupling.

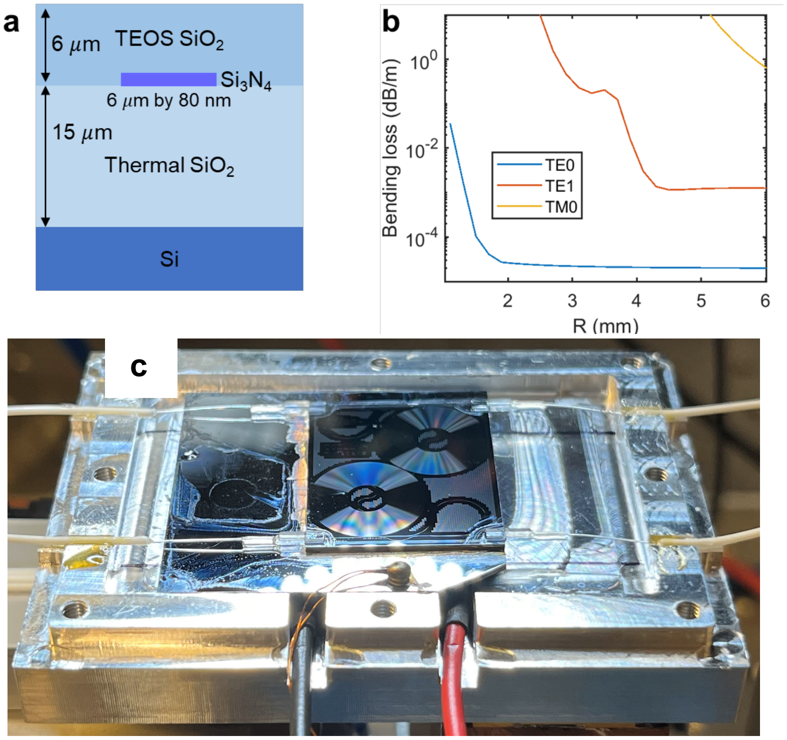

Fig. S1. Waveguide design and bending loss simulation. a The 6 μm by 80 nm thick Si_3N_4 waveguide with 15-μm-thick thermal oxide lower cladding and 6-μm-thick TEOS-PECVD deposited oxide upper cladding supports the TE_0, TE_1 and TM_0 modes at C and L bands. **b** The minimum bending radius from Lumerical MODE simulations can be as small as 1.0 mm without incurring significant bending loss in the TE_0 mode. **c** A photograph shows a coil resonator in a metal package with UHNA fibers attached to the bus waveguides and a thermal electric cooler in between the chip and the metal package for temperature regulation.

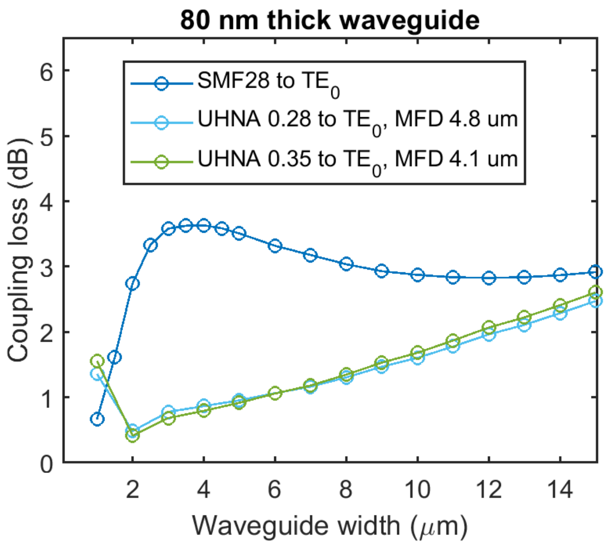

Fig. S2. Mode-overlapping and coupling efficiency simulation for the input and output waveguide with different widths and different types of fibers. The waveguide mode is simulated in Lumerical MODE at different waveguide widths and the mode-overlapping is calculated in MODE between the waveguide mode and fiber mode in different types of fibers. Note at the time of the design, the 11 μm waveguide taper width was chosen for optimizing the coupling efficiency for single-mode fiber 28 rather than the UHNA fibers and a smaller taper width such as 1.5 μm or 1.0 should be adopted in future taper designs.

The coil resonator Q and linewidth are characterized by spectral scanning the resonator using a widely tunable laser as a probe laser along with the laser detuning calibration from a unbalanced fiber MZI with an FSR of 5.87 MHz. At 1600 nm, the FSR is measured to be 48.1 MHz and the intrinsic and loaded linewidths are 1.1 MHz and 2.2 MHz, respectively, corresponding to 0.15 dB/m propagation loss (Fig. S3**a**). Such resonator Q and linewidth measurements are performed using multiple widely tunable lasers to cover the wavelength range from 1260 nm to 1650 nm (Fig. S3**b**, S3**c**).

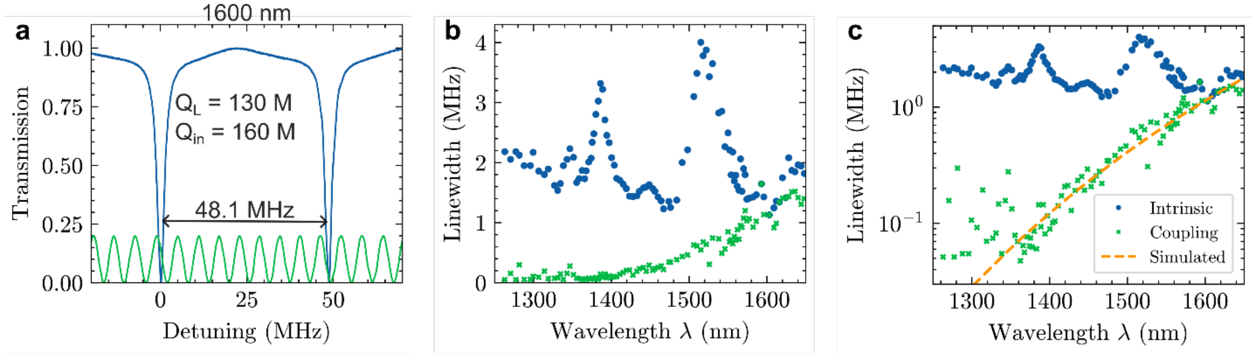

Fig. S3. Waveguide propagation loss spectrum. **a** By spectrally scanning the coil resonator using several Santec tunable lasers along with a 5.87 MHz FSR fiber unbalanced MZI for optical frequency detuning calibration, the coil resonator intrinsic loss rate γ_{in} and bus-resonator coupling loss rate γ_{ex} are measured. At 1600 nm, the waveguide propagation loss is measured to be 0.16 dB/m, the intrinsic Q is 160 million, and the FSR is measured to be 48.1 MHz. **b, c** The resonator intrinsic and coupling linewidths are measured from 1260 nm to 1650 nm and plotted in linear scale (**b**) and in log scale (**c**) and used to estimate the coil waveguide propagation loss by $\alpha = \gamma_{in} n_g / c$[5,6].

The SBS laser output from the circulator is measured on an optical spectral analyzer to ensure there is only S1 emission, increasing with the increasing pump power (Fig. S4**a**). With the calibration of fiber to chip coupling loss, and fiber connection losses, the measured S1 output power from OSA combining with the recorded pump power yield the results of the on-chip S1 power versus the on-chip pump power (Fig. S4**b**), showing the S1 threshold of 72 mW.

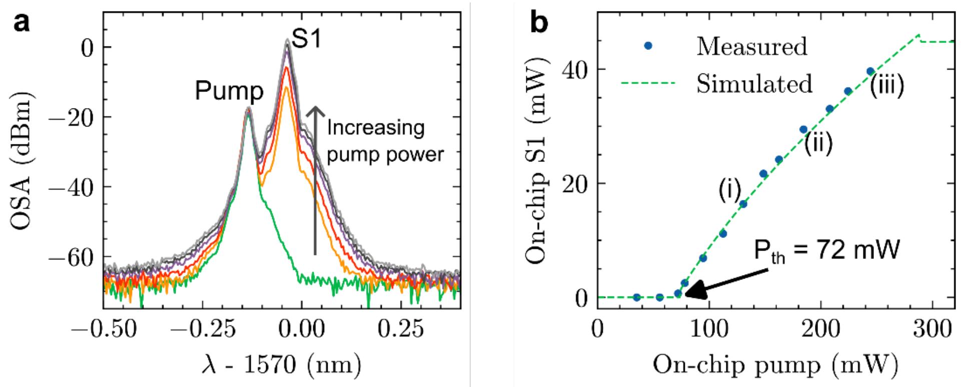

Fig. S4. Coil SBS threshold and output power. a The SBS laser S1 and pump power measured on an OSA with increasing pump power. **b** On-chip S1 power versus the pump power measures a 72 mW threshold.

In the main results, the S1 emission is recorded at 1553.3 nm, 1558.1 nm, 1563.6 nm, 1570.0 nm, 1575.8 nm, which correspond to the wavelength points where the Brillouin phase matching condition is satisfied and the S1 threshold is with the reach of the EDFA maximum output power. With the measured resonator linewidths at different wavelengths, it is possible to predict the S1 threshold at all these phase matching wavelength points using the equations in Table S1. Figure S5**a** shows the interpolation of the resonator intrinsic and coupling loss rates, which is used to calculate the S1 threshold versus wavelength (Fig. S5**b**).

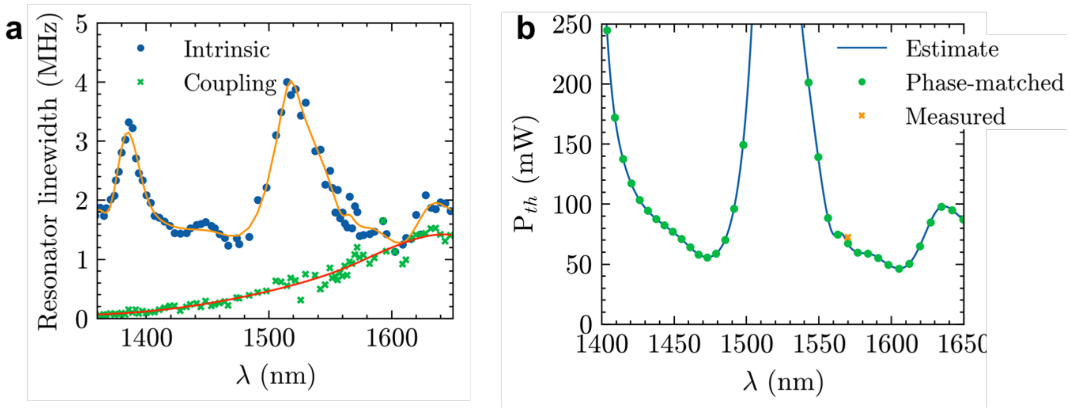

Fig. S5. Coil SBS threshold estimation at different wavelengths. a The coil resonator intrinsic and coupling loss rates are measured by a widely tunable laser and interpolated with smoothen curves. **b** Coil cavity Brillouin gain is calculated to be $\mu = 0.174$ mHz using the measured 72 mW SBS threshold at 1570 nm. Assuming the same value for μ and cavity FSR (ν_{FSR} = 48.1 MHz) at different wavelengths, using the interpolated resonator loss rates, the blue curve shows the continuous estimation of SBS threshold, and the green dots indicates the Brillouin-shift phase matched wavelengths points $\{\lambda_n : n \times \nu_{FSR} = \Omega_{ac,0}\frac{1570}{\lambda_n}\}$.

The increased resonator loss around 1520 nm incurs a significant increase in S1 threshold from 1500 nm to 1550 nm, and low threshold SBS is possible from 1550 nm to 1600 nm as well as near 1470 nm, theoretically.

Supplementary Note 2: SBS laser coupled mode model and single-mode lasing

To interpret the physics underlying the single-mode lasing displayed in Fig. 2 in the main text, we develop and analyze a coupled-mode model of SBS dynamics. This model is a generalization of prior mean-field treatments of SBS laser dynamics[1], accounting for the presence of optical modes with the potential to lase within the Brillouin gain bandwidth, and captures the complex shape of the gain spectrum by including coupling to multiple phonon modes. Owing to phase matching (distinct wave-vectors), each Stokes mode within the gain spectrum couples to distinct set of phonon modes, selected to reproduce the key features of the gain spectrum (see Fig. S6).

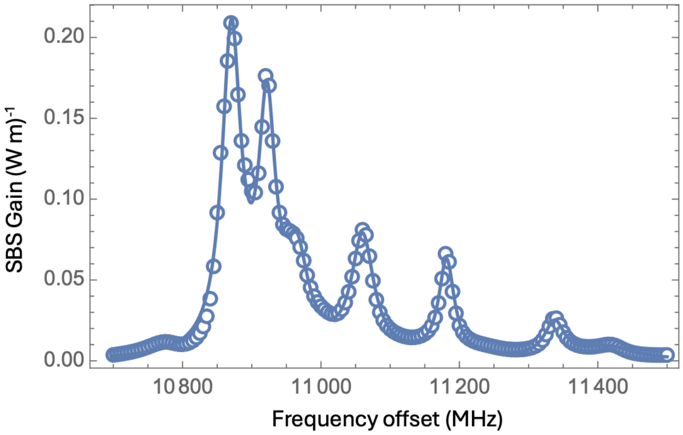

Fig. S6. Simulated and modeled Brillouin gain spectrum. Open circles show Brillouin gain spectrum simulated using finite element simulations. These simulations utilize the waveguide geometry and materials properties. The solid line represents a seven oscillator fit to Brillouin gain spectrum using parameters listed in Tab. 1.

To construct this model, we perform finite element simulations (described in Ref. [7]) to determine the Brillouin gain spectrum, and a multi-phonon model is fitted to these gain spectra to determine Brillouin coupling, frequencies and linewidths.

The Heisenberg-Langevin equations for this model are given by,

$$\dot{a}_p = -\left(i\omega_p + \gamma/2\right)a_p - i\sum_{jk} g^*{}_{jk}\, a_j\, b_{jk} + \sqrt{\gamma_{ext}}F,$$

$$\dot{a}_j = -\left(i\omega_j + \gamma/2\right)a_j - i\sum_{k} g_{jk}\, a_p b^\dagger{}_{jk},$$

$$\dot{b}_{jk} = -\left(i\Omega_{jk} + \Gamma_{jk}/2\right)b_{jk} - i g_{jk} a_p a_j^\dagger + \xi_{jk}.$$

Here, we explicitly account for coupling of multiple phonons to each Stokes mode a_j, where the coupling rates g_{jk}, frequencies Ω_{jk}, and damping rates $\Gamma_{jk}$are obtained by fitting the seven-phonon model to the simulated gain spectrum (see Tab. 1), and ξ_{jk} are Langevin forces that model thermal fluctuations of the phonons. The parameter γ_{ext} is the cavity decay rate produced transfer of energy into the bus waveguide and F is related to the supplied pump power P_{ext}through the formula $P_{ext} = \hbar\omega_p |F|^2$. The Langevin forces are assumed to be Gaussian random processes that are locally correlated in time

$$\langle \xi_{jk}^\dagger(t)\xi_{nm}(t') \rangle = \delta_{jn}\delta_{km}\,\gamma\, n_{th}\delta(t-t')$$
$$\langle \xi_{jk}(t)\xi_{nm}^\dagger(t') \rangle = \delta_{jn}\delta_{km}\,\gamma\,(n_{th}+1)\delta(t-t'),$$

where n_{th} is the thermal occupation number.

Steady-state laser dynamics

Analysis of the steady-state solutions reveals how single mode lasing can come about. In steady state, we assume that the amplitudes oscillate with constant magnitude. First, we solve for the complex phonon amplitude,

$$\mathrm{b}_{jk} = -\frac{i g_{jk} a_p a_j^\dagger}{i\Delta\Omega_{jk} + \Gamma_{\mathrm{jk}}/2},$$

where $\Delta\Omega_{jk}$ is the difference between the phonon resonant frequency and the frequency of the driving beatnote. Inserting the steady-state solution for b_{jk} into the steady-state equation for a_j we find,

$$0 = -\left(i\Delta\omega_j + \gamma/2 - \mu_j a_p^\dagger a_p\right)a_j,$$

where

$$\mu_j = \sum_k \frac{|g_{jk}|^2}{-i\Delta\Omega_{jk} + \Gamma_{\mathrm{jk}}/2},$$

and the jth mode oscillates at a frequency spaced from resonance by $\Delta\omega_j$. This equation can be solved two ways: below threshold $|a_j| = 0$, and above threshold $|a_j| \neq 0$. Above threshold the steady-state solution requires $\left(i\Delta\omega_j + \gamma/2 - \mu_j a_p^\dagger a_p\right) = 0$, giving the clamping condition for the pump,

$$a_p^\dagger a_p = \frac{\gamma}{2\mathrm{Re}[\mu_j]},$$

and the formula for the frequency offset $\Delta\omega_j = \mathrm{Im}[\mu_j]a_p^\dagger a_p = \gamma\,\mathrm{Im}[\mu_j]/(2\mathrm{Re}[\mu_j])$. The steady-state equations show that the mode with the largest Brillouin gain ($\mathrm{Re}[\mu_k] > \mathrm{Re}[\mu_{j\neq k}]$) will reach threshold first (i.e., when $a_p^\dagger a_p = \gamma/2\mathrm{Re}[\mu_k]$). At threshold the pump clamps and the only viable

solution for the remaining Stokes modes is $|a_{j\neq k}| = 0$ because $\left(i\Delta\omega_{j\neq k} + \gamma/2 - \mu_{j\neq k} a_p^\dagger a_p\right) \neq 0$.

Stochastic simulations of laser dynamics

Using finite difference method and the parameters given in Tab. 1, we simulate Heisenberg-Langevin equations to model both laser dynamics and noise. Our simulations reproduce the key features of the observed spectra and dynamics, predicting single mode lasing and large side-mode suppression. These simulation results are shown in the inset of Fig. 2 in the main text.

Table 1: Model parameters derived from finite element simulations, the parameters (g_{jk}, Ω_{jk}, Γ_{jk}) are insensitive to the Stokes mode index and assumed constant with respect to j.

Mode (k)	g_{jk}[Hz]	$\Omega_{jk}/(2\pi)$[GHz]	$\Gamma_{jk}/(2\pi)$[MHz]
0	253	10.87	33
1	189	10.93	30
2	137	10.97	40
3	156	11.07	40
4	117	11.19	27
5	82	11.27	36
6	76	11.39	90

REFERENCES

1. Behunin, R. O., Otterstrom, N. T., Rakich, P. T., Gundavarapu, S. & Blumenthal, D. J. Fundamental noise dynamics in cascaded-order Brillouin lasers. *Phys. Rev. A* **98**, 023832 (2018).
2. Yuan, Z., Wang, H., Wu, L., Gao, M. & Vahala, K. Linewidth enhancement factor in a microcavity Brillouin laser. *Optica* **7**, 1150–1153 (2020).
3. Li, J., Lee, H., Chen, T. & Vahala, K. J. Characterization of a high coherence, Brillouin microcavity laser on silicon. *Opt. Express* **20**, 20170 (2012).
4. Liu, K. *et al.* Integrated photonic molecule Brillouin laser with a high-power sub-100-mHz fundamental linewidth. *Opt. Lett.* **49**, 45–48 (2024).

5. Puckett, M. W. *et al.* 422 Million intrinsic quality factor planar integrated all-waveguide resonator with sub-MHz linewidth. *Nat. Commun.* **12**, 934 (2021).
6. Spencer, D. T., Bauters, J. F., Heck, M. J. R. & Bowers, J. E. Integrated waveguide coupled Si_3N_4 resonators in the ultrahigh-Q regime. *Optica* **1**, 153 (2014).
7. Gundavarapu, S. *et al.* Sub-hertz fundamental linewidth photonic integrated Brillouin laser. *Nat. Photonics* **13**, (2018).
